# Why Adolescents Participate in a Music Contest and Why They Practice – The Influence of Incentives, Flow, and Volition on Practice Time

**DOI:** 10.3389/fpsyg.2020.561814

**Published:** 2020-10-27

**Authors:** Claudia Bullerjahn, Johanne Dziewas, Max Hilsdorf, Christina Kassl, Jonas Menze, Heiner Gembris

**Affiliations:** ^1^Department of Social Sciences and Cultural Studies, Institute of Musicology and Music Education, Justus-Liebig-University Giessen, Giessen, Germany; ^2^Faculty of Cultural Studies, Institute for Research on Musical Ability, Paderborn University, Paderborn, Germany

**Keywords:** music contests, adolescents, musical practice, motivation, incentives, flow, volition, musical genres

## Abstract

Music contests are a means of discovering talents and promoting musical abilities. Participation in a contest is usually preceded by many years of practice requiring a high level of motivation and a supportive environment, especially regarding family. Despite the importance participation in music contests may have for musical development, there is a considerable research deficit. The annual music contest “Jugend musiziert” (youth making music) is the most important musical competition for highly gifted young musicians in Germany. There has been comprehensive research on the participants of “Jugend musiziert” by Hans Günther Bastian in the 1980s and 1990s, but since then, only very little research has been published. In 2017, we started a large-scale study on the participants at the national level, covering a broad range of topics, including sociocultural background, development and learning, performance practice, personality traits, motivation, and musical performance anxiety. A standardized paper-pencil questionnaire was administered to approximately 2,260 participants and a total of 1,143 valid questionnaires was returned (age 9–24 years; *M* = 15; *SD* = 2.1, female = 62%). Using principal component, variance, correlation, and linear discriminant analyses, interdependencies between practice time and motivational factors were analyzed in this paper. Concerning practice time, major differences between participants of different contest categories became clear, with classical musicians practicing the most. Practice time, as well as parental support and supervision, correlated with age: Older participants spent, on average, more hours practicing and received less support and supervision. Challenge was the most important incentive for all participants, but more decisive for participants in the classical solo contest than in the ensemble category. Female participants were more prone to fear incentives than males. Participants who practiced a lot scored higher on general flow than the participants with a smaller amount of practice and also showed significantly more perseverance. Moreover, participants of the pop solo contest experienced more general flow than all other participants; ensemble players showed more social focus than participants in the classical solo contest. All in all, participants of different contest categories could be discerned by practice time and prototypical motivational aspects.

## Introduction

### “Jugend musiziert” and Practice Time

The “Jugend musiziert” music competition has been the largest, the most important and influential music contest for young musicians in Germany for over 50 years. It is under the patronage of the Federal President of the Federal Republic of Germany and is sponsored by the Federal Ministry for Family Affairs, Senior Citizens, Women and Youth, the NGO “Deutscher Musikrat” (German Music Council), as well as several other sponsors, such as financial institutions. This differs considerably from the contests in other parts of the world, which are often supported by music teacher associations or foundations. Traditionally, the contest has been geared toward young classical musicians. However, the competition has also recently begun to include popular music. Its main aims are the encouragement of young musicians, as well as promoting musical talents, and encouraging them to choose a career as a professional musician. “Jugend musiziert” is organized in three levels. Every participant first performs at the regional competition. If the participants score at least 23 out of 25 possible points, they are promoted to the federal state level, at which the entire procedure is repeated. The final and highest level is the national level. Participants at the national level of the contest are commonly regarded as highly gifted young musicians. It is at this level that our study was conducted.

There are several reasons why it is valuable to examine “Jugend musiziert” more closely. Firstly, the sample the “Jugend musiziert” contest presents at the national level is unique because it attracts a lot of high-achieving, highly motivated and highly gifted adolescents. Secondly, a study of the “Jugend musiziert” contest offers insights into the feelings, goals, and ambitions of adolescents during the situation of the contest, which have been rarely examined before. Thirdly, due to the use of standardized instruments, this study takes a more objective approach than most of the other studies on “Jugend musiziert” conducted previously. Lastly, the motivational aspects related to the “Jugend musiziert” contest have not been focused on in detail. Therefore, we included individual aspects such as incentives, flow, and volition in our study. Additionally, our large-scale, superordinate survey sought to provide information regarding reasons for student participation in the contest and other relevant aspects, such as personality traits and performance anxiety.

Research on “Jugend musiziert” and its participants is scarce. About 30 years ago Bastian(1987; 1989; 1991, follow up: Bastian and Koch, 2010) conducted several comprehensive research projects on the participants of the contest using narrative interviews and questionnaires to investigate the attitudes of the young participants toward the contest as well as their emotions and their thoughts both preparing for the contest and during their performances. He also included opinions and statements from parents, organizers, and members of the jury. Previous research on “Jugend musiziert” has rarely covered motivational aspects with only few exceptions (Linzenkirchner and Eger-Harsch, 1995; Bullerjahn et al., 2017). Austin (1988) compared the effects of two music contest formats (rated vs. comments only) for fifth- and sixth-grade band students on self-concept and achievement motivation, amongst other things. The results showed significant gains in musical self-concept for both groups, but only rated students experienced significant gains in achievement motivation scores. Gouzouasis and Henderson (2012) examined the educational, musical and social benefits and detriments that evolve from participation in a competitive band festival. They found that personal beliefs and feedback from their directors, adjudicators, parents, and peers influenced the attitudes of students on how they perform. Furthermore, students appreciated the competitive aspects of music festivals as a motivational factor when practicing and performing and as a possibility for developing a sense of pride after accomplishing good performances. For young participants in international pop singing competitions, higher performance quality was associated with positive emotions, low arousal and increased dominance, while lower performance quality was associated with negative emotions (Rucsanda et al., 2020). Also, “experiencing positive emotions before a competition could be a significant predictor of success” (ibid., p. 489). However, solo contests in general seem to be more stressful for students than small ensemble or band contests, especially for females, but were perceived as having the most motivational value (Howard, 1994).

Since preparing for a music competition like “Jugend musiziert” requires a lot of practice, be it alone, with a teacher, or together in an ensemble, the participants at the national level of the contest can be considered as having a high musical expertise compared to other adolescents. Expert performance is generally explained by the accumulated amount of deliberate practice (Ericsson et al., 1993; Gruber and Lehmann, 2014). However, expertise development theories have been based on the expertise development of classical musicians, suggesting that expertise is developed over about 10 years and that formal instruction, formal practice, and parental support are crucial for expertise development. Moreover, it should be noted that practice time only refers to the “quantity of time devoted to practice, which can be more or less formal” (Bonneville-Roussy and Bouffard, 2015, p. 689). In particular, popular music practice on its prototypical instruments (e.g., drum set, electric guitar), which had been shown to be more informal, has not received much attention, especially concerning motivational aspects (Wissner, 2018).

In earlier studies, the average practice time of participants of “Jugend musiziert” on federal state and national level was found to be approx. 16 h per week (Linzenkirchner and Eger-Harsch, 1995) and 24 h per week (Bastian, 1991). Unfortunately, it remains unknown whether musical practice ahead of the contest met all criteria of deliberate practice. In prior studies, the role of motivation has often been grossly underestimated, despite the tremendous impact it has on both quantity and quality of practice. Thus, it should be noted that motivation constitutes a very important component of time that individuals spend practicing and rehearsing.

### Motivation as an Umbrella Term

Motivation is an umbrella term for incentives, motives, flow, and volition. Unfortunately, the field of motivational research is rather disconnected. The present study concentrates on the models and theories by Heckhausen and contributors because they are, to our knowledge, the only ones which include all relevant aspects listed above. Incentives are motivational factors offered by a certain situation whereas motives are motivational factors within people themselves. Incentives are defined by Heckhausen and Heckhausen(2018, p. 6) as follows: “Every positive or negative outcome that a situation can promise or signal to an individual is called an ‘incentive’ and has ‘demand characteristics’ for an appropriate action.” Combined, motives and incentives define the affordances of a situation or activity (Heckhausen and Heckhausen, 2018, pp. 1–14). The more one expects their actions to positively influence the outcome of a situation, the more incentivizing it is (Rheinberg, 1989). For highly developed motives, only little incentivization is needed to foster motive-related actions (Roth, 2012). While incentives can be regarded as activity-based or outcome-based, a combination of both is common among creative and artistic activities (Roth, 2019). In the case of the present study, the “Jugend musiziert” contest represents the situation through which the participating adolescents become motivated, which is what is meant by the concept of incentives. Roth (2012, 2013, 2019) was the first to systematically study the incentives for musical practice. Against the backdrop of several findings, it was argued “that motivation is a greater predictor of practice than vice versa” (Bonneville-Roussy and Bouffard, 2015, p. 698).

Another well-established cognitive approach to motivational research in music education is the Expectancy-Value Theory (Eccles, 2005), focusing on both musicians’ expectations and the value attributed to music-related activities, such as a graded music performance examination. Instrumental music learners’ judgments concerning the likelihood of success in the music examination were positively associated with the results they actually obtained and, therefore, can be considered powerful predictors of achievement (McCormick and McPherson, 2007).

The two main lines of motivation research around Self-Determination-Theory (SDT) (Deci and Ryan, 1985; Ryan and Deci, 2017) and Motive-Disposition-Theory (MDT) (McClelland, 1985) have both established a three-factor-model of motives. While SDT’s needs for competence and relatedness are similar to MDT’s needs for achievement and affiliation, respectively, the needs for autonomy (SDT) and power (MDT) differ. The competence or achievement motive is characterized by the commitment to a “standard of excellence” and the pursuit of “achievement goals on one’s own initiative” “to excel oneself” or “to rivel or surpass others” (Brunstein and Heckhausen, 2018, p. 221) and to avoid feared failure (cf. ibid, p. 228-230). The affiliation or relatedness motive describes “the fundamental human need for social acceptance, belonging, and interpersonal exchange” (Hofer and Hagemeyer, 2018, p. 306) as well as “the strong fear of social rejection and isolation” (ibid., p. 313). The power motive is the desire to influence “the physical states, thoughts and/or emotions of other people” (Busch, 2018, p. 338). “The central incentive of the power motive is the experience of strength and social impact,” but also the “fear of weakness,” thus promoting independence and autonomy (ibid., p. 338). In contrast, the autonomy motive is only interpreted as “the need to self-regulate one’s experiences and actions […] associated with feeling volitional, congruent, and integrated” (Ryan and Deci, 2017, p. 10).

While the study on hand follows MDT’s terminology and conceptualizations, other researchers, especially in Anglo-American countries, adapted the SDT-approach to further investigate motivation. Evans (2015) utilized the SDT-approach to motivation in music education in order to unify the various theoretical approaches used in music education in an umbrella approach. He also provided an overview of music motivation studies, which support this meta-theory. The Motivation and Engagement Wheel (Martin et al., 2016) is a proposal to amalgamate motivation and engagement, i.e., the behavior that follows from this motivation, in an integrative multidimensional model.

In the substantial body of research on motivation, the distinction between self-determined motivation, associated with fulfilling the action required for the task (“intrinsic”), and the drive to perform a task because there is an association with a particular outcome outside the task (“extrinsic”) is widespread (Covington and Müeller, 2001; Heckhausen and Heckhausen, 2018, p. 6). It was found that intrinsic motivation increases with level of music activity, with professional musicians scoring higher than amateurs. Furthermore, females have more intrinsic motivation than males (Appelgren et al., 2019).

In recent years, interest in flow as another component of the broad term “motivation” within music contexts has increased tremendously (Tan and Sin, 2019). The concept of flow, originally described by Csikszentmihalyi, refers to a state which is characterized by the following components: (a) focusing exclusively on one’s actions in the present, (b) a sense of control over one’s actions, (c) experiencing the activity as pleasurable, (d) an absence of self-consciousness, as well as (e) a distorted perception of time, and (f) “the merging of action and awareness” (Nakamura and Csikszentmihalyi, 2005, p. 90). Flow can only occur if certain prerequisites are fulfilled: firstly, that the demands of a situation and an individual’s skills are properly matched; and secondly, that clear goals and immediate feedback are provided (Nakamura and Csikszentmihalyi, 2005).

Flow has been demonstrated to increase with age. Furthermore, a negative correlation between flow and musical performance anxiety was determined (Cohen and Bodner, 2019). Consequently, it is surprising that no correlation between gender and flow was found, as there is a gender effect with regard to musical performance anxiety (ibd.). It has been shown that experiencing flow is vital to ensuring continued interest and participation in musical activities (Lamont, 2012). Therefore, it is not unexpected that flow has been found to be positively correlated with the total amount of practice time (Butkovic et al., 2015) and self-regulation (Araújo and Hein, 2016). Additionally, moderate-achieving teenage musicians experienced flow less often than high-achievers from a specialist music school (O’Neill, 1999). Furthermore, flow experiences seem to be facilitated by trait emotional intelligence, specific structural and compositional features of musical pieces as well as related emotional expressions (Marin and Bhattacharya, 2013). A relationship between personality and the ability to experience flow was also established for amateur singers with extraversion correlating positively with flow experiences, while neuroticism exhibited a negative correlation with flow experiences (Heller et al., 2015).

Apart from incentives and aspects of flow, volitional factors are also of great importance regarding the practicing behavior of the participants. Volition describes aspects of will that can be helpful in maintaining certain actions and behavior even if inner resistance is present or motivation is low (Sokolowski, 1993). As opposed to actions which are propelled by high motivation, actions that involve a great amount of volition are perceived as exhausting. People experience less fun during their actions and time seems to pass very slowly (Roth and Sokolowski, 2011).

Up until today, volition has mostly been investigated in the area of psychology and implemented in general models of action and behavior (Heckhausen and Gollwitzer, 1987; Heckhausen and Heckhausen, 2018). Research on volition during the practice of a musical instrument has been scarce. Volition has been investigated in combination with motivation and the maturing process, showing an influence of motivation and volition on the practicing methods (Harnischmacher, 1998). During practice, volitional components and strategies seem to help in maintaining the practice process even if one does not want to practice (Roth, 2012). There has been research on the connection between flow and volition, stating that flow can occur even if one practices with reluctance. This can be explained by either a transition from a volitional to a motivational mindset during practice (Roth and Sokolowski, 2011) or the automatic occurrence of volitional processes during a period of high motivation (Kehr, 2004).

As stated above, motivation with its components incentives, flow, and volition has rarely been investigated in the context of music competitions. Hence, this paper attempts to enrich the state of research in this regard.

### Aims and Research Questions

One main aim of the superordinate large-scale study is to acquire basic information about the participants of “Jugend musiziert,” their living environment, and sociocultural background. Another goal of this research project is the further exploration of the role of music and the contest itself in the lives of the participants. Additionally, updating and expanding on earlier findings is also an important aim. The superordinate as well as this specific part of the study are explorative in nature, as most of the prior studies were qualitative (a notable exception being Mund, 2007) and did not rely on established standardized testing methods. Although the different aspects of this study have been examined individually before, this large-scale study is the first one, at least to our knowledge, to combine all these aspects into one study. Clearly, this is advantageous as all aspects were examined using the same testing methods and sample, allowing for higher levels of comparability of results regarding the different aspects examined.

This specific paper will concentrate on practice time as well as motivation and examines different motivational aspects: the incentives for participating in “Jugend musiziert,” flow as an incentive while practicing, and volition during practice as well as during the audition in front of a jury. Therefore, we will investigate

(a) Which incentive, volition and flow factors exist in the participants of “Jugend musiziert”,
(b) Whether and how participants of different contest categories differ concerning their amount of practice,
(c) Whether and how participants of different contest categories differ concerning incentive and volition factors as well as factors of flow as reasons to participate in the contest, and
(d) Whether it is possible to establish a typology by comparing classical solo players, classical ensemble players, and pop solo players.

## Materials and Methods

### Procedure

Our survey was conducted in 2017 at the national contest of “Jugend musiziert” in Paderborn (see Gembris and Bullerjahn, 2018, 2019 for more details). That year, 20,529 adolescents participated at the regional level, 8,300 at the federal state level, and 2,732 at the national level, which means that a little more than 10 percent of all participants made it to the national level. These are the official numbers that include double participation of musicians in different categories of the contest. In 2017, the “Jugend musiziert” contest included the following instruments and categories (instrument categories change every year):

• Solo instruments: piano/harp/voice/drum set (pop)/guitar (pop).
• Ensembles: strings/winds/chamber music for accordion/ “Neue Musik” (New Music; i.e., avant-garde music of the 20^th^ century/contemporary music)^1^.

### Measures

In our survey, we used a standardized paper-pencil questionnaire (17 pages) including some open questions, which were distributed to approximately 2,300 participants in person. Existing standardized instruments and items were integrated into our questionnaire: items concerning the incentives for participation in the contest (Bullerjahn et al., 2017), items of the Flow Short Scale (“Flow-Kurzskala”; Rheinberg et al., 2003), items for playing-related disorders (Gembris and Ebinger, 2017), the German version of the Music Performance Anxiety Inventory for Adolescents (MPAI-A-D) (Osborne and Kenny, 2005; unpublished German translation used in Nusseck et al., 2015), items from the sport-specific Volitional Components Questionnaire, adapted for musical practice (VKS; Wenhold et al., 2009), items concerning the attitude toward practice, parental support and the use of media (Krupp-Schleußner, 2016), and the 10 Item Big Five Inventory (BFI-10) (Rammstedt et al., 2013). All answers were captured by a 5-point Likert scale (from “strongly disagree” to “strongly agree”).

Due to the overall length of the questionnaire and the special sample of the contest participants, some items had to be excluded and the existing instruments had to be adapted to form a fitting instrument for the “Jugend musiziert” contest. Some items were considered to offer little additional information; for example, it was decided to exclude an item that asked whether participants wanted to score higher than most others, as compared to an item asking about whether winning a prize was important to the participant. For the incentive measurement, the aim was always to include at least two items that expressed possible hopes and two that expressed possible fears of the contestants and this concerning every need. Overall, because of the rare opportunity to explore a sample comprised of the national level participants of “Jugend musiziert,” it was determined to gather as much information as possible. This was achieved by covering a broad variety of aspects in one questionnaire.

### Participants

1,143 valid questionnaires were filled in and returned by the participants, which means that the rate of return was approximately 50 percent. The majority of the participants returning their questionnaire were female (*n* = 692, 62%); 38 percent were male (*n* = 427). The participants’ age ranged from 9 to 24 years (*M* = 15.08, *SD* = 2.14).^2^ Thus, a vast majority was still in school. There were no gender differences regarding the age distribution.

Participants were unevenly distributed across the different contest categories. In our sample, 70 percent participated in one of the ensemble contests (strings, winds, accordion, “Neue Musik”), 25 percent performed in one of the classical solo contests (piano, harp, voice), while only 3 percent of the sample were part of the pop solo contests (drum set, guitar). Accompanists made up 2 percent of the sample. Some participants took part in both the ensemble contest and a solo contest (1%). Table 1 shows selected descriptive statistics about the sociodemography of the participants in each of the three contest categories. Comparing the three different categories, “classical solo,” “pop solo,” and “classical ensemble,” some sociodemographic differences could be found. Whereas the classical categories showed a higher number of female participants, the number of male contestants in the pop solo categories surpassed the number of females by far. Concerning the occupation of the contestants, there were only small differences between the groups. Across all groups, most of the participants showed a high level of education.

**TABLE 1:** Sociodemography of the participants in the three contest categories.

		Classical solo (*n* = 280)	Pop solo (*n* = 38)	Classical ensemble (*n* = 795)
Age	Mean	15.39	15.03	14.94
	*SD*	2.26	2.07	2.07
Gender	Male	41.67%	86.49%	34.19%
	Female	58.33%	13.51%	65.56%
Occupation	Gymnasium*	82.40%	86.84%	85.10%
	Other type of school	10.86%	10.50%	10.00%
	Apprentice or employed	1.49%	2.63%	0.39%
	University**	5.20%	0.00%	2.07%
Immigration background***	Personal	7.97%	7.89%	3.60%
	Only Parental	35.14%	18.42%	16.48%
	None	56.86%	73.68%	79.92%
Parental profession****	Academic profession	51.43%	57.89%	57.36%
	Music related profession	23.93%	15.79%	22.26%
Residence	Big town/city	26.26%	13.16%	22.40%
	Medium size town	27.70%	34.21%	37.00%
	Small town/village	46.04%	52.63%	40.63%
Number of siblings	Mean	1.44	1.34	1.65
	*SD*	1.14	1.17	1.18
Individuals motivating contestants to participate	Parents	34.29%	26.32%	24.28%
	Instrumental teacher	72.14%	65.80%	77.11%
	Self-encouraged	67.50%	47.37%	49.43%
	Others (e.g., siblings and peers)	8.57%	7.89%	19.50%
Teacher chosen for preparation for the contest	Teacher at a music school	46.77%	71.95%	60.41%
	Private music teacher	21.22%	13.16%	14.75%
	College professor	14.75%	2.63%	9.06%
	No teacher (autodidact)	0.36%	2.63%	0.26%
	More than one teacher	9.72%	10.53%	10.22%

*In Germany, students are traditionally placed in one of three types of schools (Hauptschule, Realschule, and Gymnasium) after fourth grade. The Gymnasium is meant to prepare above-average students for university studies. **University students can only participate in the contest if they are not in any music related programs. ***Immigration background was only tracked up until the parents’ generation. Earlier immigration history is not included and assumed to be non-existent. Concerning 2017, the Federal Statistical Office for Germany reported an immigration background in 23.6% of cases (cf. Destatis, 2018, p. 41). ****Values describe the percentage of participants with one or both parents in the specified profession.

Students received musical education concerning their musical instrument or voice respectively in a variety of contexts. 57 percent were taught in music schools, 16 percent by a private teacher. Also, there is a distinction between private music schools (6%) and VdM music schools (51%; VdM = abbreviation for German Music School Association). Although VdM music schools are not part of the public education system, these music schools teach according to standardized curricula, whose organization roughly aligns with the general education system. The VdM does not view its music schools as child care centers. Rather, they consider themselves educational institutions. Their focus is on both continuity and care. Furthermore, some students were educated by college professors (10%). Another 10 percent reported having more than one teacher; only 0.4 percent were autodidacts. It is intriguing that participants of both classical categories were trained by private teachers or college professors more often than pop solo participants. However, the largest share of participants across all contest categories were taught in music schools. For all categories, except for classical solo, this share was larger than 50 percent (cf. Table 1).

Although more than half of the participants of all contest categories stated they did not have an immigration background, the classical solo contestants, when compared to the other two groups, included a large number of contestants whose parents immigrated to Germany. In all three categories, most of the participants stated coming from a small town or village. Regarding the participants’ parents, it is striking that – across all three categories – more than half of the parents worked in an academic profession, showing that most of the participants come from a high-level educational background. It is also noteworthy that fewer parents of pop solo contestants worked in a music related profession, when compared to the two classical categories (cf. Table 1).

### Data Treatment and Analyses

All data were analyzed using the IBM Statistical Packages for the Social Sciences (SPSS 27). Data visualizations were created using Python 3.7.6 with the packages Matplotlib 3.1.3 and Pandas 1.0.1. Explorative principal component analyses were computed so that our scope was not limited to looking at single items but also included latent variables. In order to answer the research questions, we mainly used analyses of variance. Correlations were computed to test for coherences between the newly built scales. Furthermore, a linear discriminant analysis (LDA) was conducted, so differences between participants in the three contest categories could be investigated further.^3^ The level of significance was set at *α* = 0.05.

## Results

### Amount of Practice, Attitude Toward Practice, Requirements Fit, and Support by Parents

**FIGURE 1:**
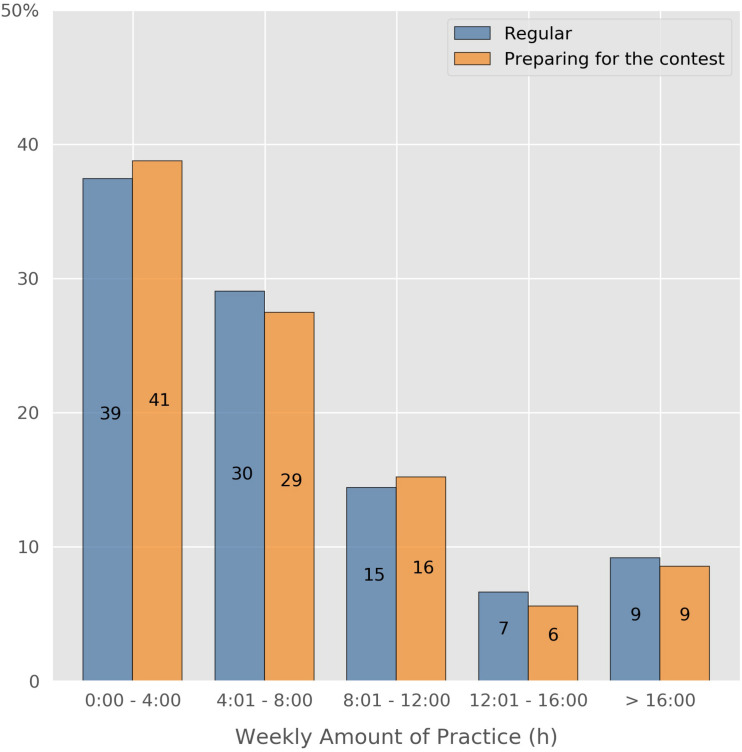
Regular weekly amount of practice in comparison to weekly amount of practice in preparation for “Jugend musiziert”.

We asked the participants about their regular daily practice time, as well as the number of days per week they usually practice.^4^ Weekly practice time was then calculated by multiplication. The practice time in preparation for “Jugend musiziert” was determined in the same manner. In both cases, participants practiced about 7 h per week on average (regular practice time: *M* = 7:03 h, *SD* = 6:31 h; in preparation for “Jugend musiziert”: *M* = 7:10 h, *SD* = 6:17 h) (cf. Figure 1). The small differences between regular practice time and practice time in preparation for the contest may be explained by two contrary strategies: 48 percent of the participants increase their practicing efforts before the contest, while 35 percent reduce it. One possible explanation for this phenomenon could be that, since participants play the same pieces at the different stages of the contest, it may not appear necessary to increase practice time for the national level contest because they are already capable of playing their pieces very well. 17 percent report no changes to their practice time before the contest.

**FIGURE 2:**
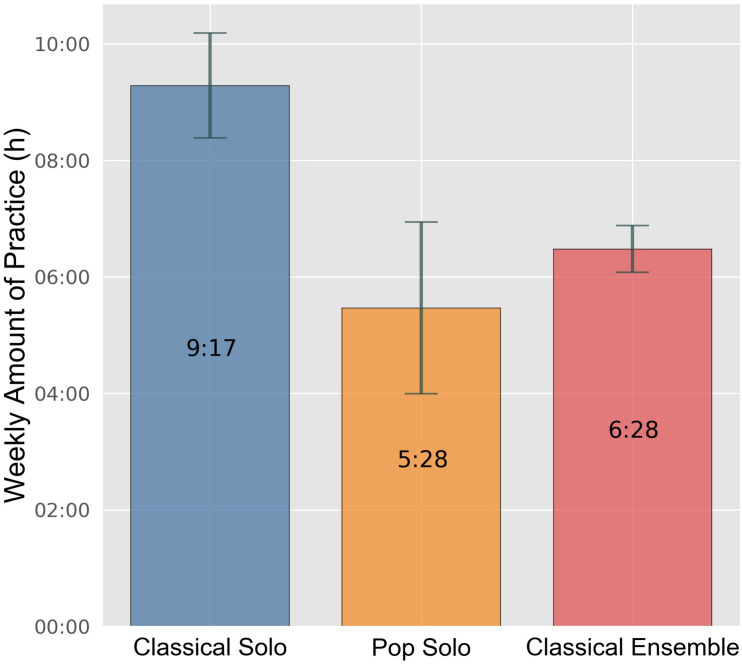
Mean regular weekly amount of practice by contest category. Error bars indicate 0.95 confidence interval estimations (standard error * 1.96).

There were no significant differences between male and female participants. However, major differences can be identified between participants of the classical solo contest category compared to the pop solo and the ensemble contest category: Participants in the classical solo contest category usually practiced about 9 h per week, and, therefore, significantly more than participants of other contest categories, who averaged about 6 h weekly (Welch-ANOVA *F*[2,97.691] = 17.353, *p* < 0.001), although high standard deviations for the amount of practice within the groups could be observed (cf. Figure 2). In preparation for “Jugend musiziert,” participants in the classical solo contest category even practiced, on average, about 11 h weekly, whereas participants in the ensemble contest category practiced a little bit less than before (Welch-ANOVA *F*[2,92.208] = 52.283, *p* < 0.001).

Age also has a significant influence on the amount of weekly practice: while there is no correlation between the age of the participants and the time spent practicing while explicitly preparing for the contest, regular practice time correlates with age. It is not surprising that, generally, it are the oldest participants who spend the most hours practicing. However, the remarkably high standard deviations indicate that there are some musicians, even among the highest age cohort, who usually spend relatively little time practicing (Welch-ANOVA *F*[3,469.630] = 8.183, *p* < 0.001). In particular, this holds true for participants in the classical solo contest. In any case, we can observe considerable inter-individual differences in practice time (cf. Table 2). Both “amount of practice” variables approximate a Pareto distribution (see Supplementary Figure 2).

**TABLE 2:** Practice related variables for different contest categories, age groups, and genders.

		Contest category			Age				Gender	
		Classical solo *n* = 280	Pop solo *n* = 38	Classical ensemble *n* = 795	9–13 *n* = 293	14–15 *n* = 362	16–17 *n* = 318	18–24 *n* = 150	Female *n* = 692	Male *n* = 427
Amount of practice per week	High amount of practice*	43.12%	18.92%	27.30%	25.61%	30.84%	31.09%	42.76%	30.79%	32.13%
	Mean (in h)	09:17	05:28	06:28	06:02	06:49	07:38	09:16	07:03	07:23
	*SD* (in h)	07:31	04:34	05:44	04:44	05:47	06:43	08:26	06:14	06:26

Perceived	Mean	3.52	3.44	3.46	3.43	3.53	3.50	3.43	3.51	3.43
challenge from the contest	*SD*	0.61	0.61	0.60	0.55	0.60	0.65	0.63	0.59	0.63

Parents come to	Mean	4.58	4.54	4.56	4.66	4.60	4.55	4.28	4.57	4.54
concerts	*SD*	0.69	1.01	0.70	0.54	0.72	0.70	0.89	0.66	0.79

Parents invest time	Mean	4.90	4.86	4.87	4.90	4.88	4.89	4.78	4.88	4.86
and money	*SD*	0.34	0.35	0.44	0.33	0.39	0.38	0.62	0.40	0.44

Positive attitude	Mean	4.22	4.31	4.02	4.12	4.07	4.09	4.06	4.09	4.07
toward practice	*SD*	0.69	0.83	0.77	0.68	0.79	0.79	0.76	0.71	0.82

Age starting instrumental lessons	Mean	7.49	6.83	7.25	6.16	7.08	7.66	9.15	7.29	7.27
	*SD*	3.41	2.27	2.49	1.91	2.37	2.78	3.60	2.84	2.58

Except for the amount of practice and age starting instrumental lessons, means indicate values on a 1–5 Likert scale. *Participants with more than 8 h of practice were assigned to “high amount of practice”.

Contestants were assigned to the groups “high amount of practice” and “low amount of practice” based on their regular practice time. The threshold was set at 8 h per week. The overall attitudes toward practice and parental support were measured with items taken from a relatively recent nation-wide survey (Krupp-Schleußner, 2016). Table 2 shows differences concerning practice-related variables for different contest categories, age groups, and genders. Regarding the number of contestants who stated a high amount of practice, huge differences between the contest categories could be established. Whereas about 40 percent of participants in the classical solo contest practiced more than 8 h per week, only about one fifth of the contestants reported this amount of practice in the pop solo category. Compared to the other two categories, participants of the classical solo category also showed a higher amount of practice measured in hours per week in preparation for “Jugend musiziert,” practicing about 3–4 h more than contestants of the other categories (Welch-ANOVA: *F*[2,92.208] = 52.283, *p* < 0.001). In addition, the amount of practice rose with increasing age. The older the participants, the more they practiced.

As a general rule, all participants received substantial support by their parents, measured by the parents’ willingness to invest time and money in instrumental lessons and to attend their children’s concerts (cf. Table 2), although it becomes clear that parents come to their children’s concerts more often when the children are young. Also, most of the participants showed a positive attitude toward practicing in general and excelling on their instrument. However, only the minority of the participants got parental supervision in form of being prompted to practice regularly and receiving help when experiencing practice difficulties. Interestingly enough, young musicians prompted to practice by their parents do not show a significantly higher amount of time spent practicing. Parental support and supervision correlate negatively with age, showing that older participants received less support and experienced less supervision by their parents. Additionally, contestants in the classical ensemble category seem to have a slightly less positive attitude toward practicing than the other two groups. Across all these practice related variables, no gender differences could be found.

49 percent of the participants at the national level rated the demands of “Jugend musiziert” as exactly right, while 48 percent felt either challenged or even overtaxed, and therefore, possibly did not experience flow. There were no major differences between the contest categories (cf. Table 2: “perceived challenge from the contest”).

### Validity and Reliability of the Used Scales

After the survey, we conducted factor analyses to assure the construct validity of our scales. Furthermore, we calculated correlations with the personality factors of the BFI-10 that confirmed the criterion validity of our instrument.

A principal component analysis was computed for the twelve selected items taken from the questionnaire on incentives for participating in “Jugend musiziert” (Bullerjahn et al., 2017). In

its original version, the questionnaire was designed to represent the three basic psychological needs of power, achievement, and affiliation, as well as flow and volition. Only four items, each representing the basic psychological needs, were used in this study. However, in the end only two factors could be interpreted as internally consistent constructs. These were then transformed into two scales (“fear” [Cronbach’s α = 0.630] and “hope for affiliation” [Cronbach’s α = 0.666]). The two statements left (“I take part in the contest as a personal challenge” [“challenge”] and “I want to be admired for my performance” [“hope for admiration”]) were included as single items (cf. Table 3).

**TABLE 3:** Results of the factor analysis for the incentive items.

			Hope for	Third factor
Items		Fear	affiliation	(not interpreted)
I fear performing badly.		**0**.**788**	–0.044	0.039
I fear playing or singing worse during the audition than during practice.		**0**.**747**	–0.075	–0.108
I don’t want to disappoint anyone with my playing.		**0**.**586**	0.135	0.066
I fear not getting the amount of attention I deserve.		**0**.**493**	–0.056	0.431
I fear friendships breaking apart due to my participation in the contest.		**0**.**441**	0.128	0.074
I fear giving away too much of myself by singing or playing emotionally.		**0**.**401**	0.059	0.122
I participate to make friends.		0.118	**0.864**	–0.028
I participate to meet other musicians.		0.048	**0.858**	0.016
I participate to move others with my music.		–0.021	**0.498**	0.432
Comparing myself to other participants helps me to improve my playing or singing.		0.077	**0.457**	0.341
I want to be admired for my performance.		0.203	–0.040	**0.767**
I take part in the contest as a personal challenge.		–0.014	0.200	**0.646**
	Cronbach’s *α*	0.666	0.630	0.346
	Variance explained (%)	18.20	16.92	12.78

*Extraction method: principal component analysis (PCA). Rotation method: Varimax with Kaiser-Normalization. KMO: 0.687, Bartlett: *p* < 0.001. Bold values indicate the highest factor loading for each item.*

We only utilized nine items from the Flow Short Scale by Rheinberg et al. (2003). A principal component analysis resulted in two main factors, in line with the original inventory: “concern” (Cronbach’s α = 0.555) and a “general flow” factor (Cronbach’s α = 0.742) were identified (cf. Table 4).

**TABLE 4:** Results of the factor analysis for the flow items.

Items		General flow	Concern
During the entire practice session, I feel like I know exactly what to do.		**0.746**	–0.052
During practice, I am completely drawn into the activity.		**0.709**	–0.189
During practice, I feel in control of my practice schedule.		**0.667**	0.108
During practice, time flies.		**0.638**	–0.008
My practice is moderately demanding.		**0.605**	–0.120
During practice, my thoughts and activities flow smoothly.		**0.582**	–0.029
During practice, I worry about failure.		0.161	**0.772**
During practice, something important is at stake.		–0.241	**0.721**
I must not make any mistakes during practice.		–0.068	**0.623**
	Cronbach’s *α*	0.742	0.555
	Variance explained (%)	30.06	17.44

*Extraction method: principal component analysis (PCA). Rotation method: Varimax with Kaiser-Normalization. *KMO*: 0.746, Bartlett: *p* < 0.001. Bold values indicate the highest factor loading for each item.*

Eight items from the Volitional Components Questionnaire (VKS) by Wenhold et al. (2009) were selected and a principal component analysis was computed. Unfortunately, Cronbach’s Alpha was found to be mediocre. In particular, the first factor “social focus” (Cronbach’s α = 0.431) was found to be quite inconsistent due to one item. The second factor “perseverance” was slightly more consistent (Cronbach’s α = 0.611) (cf. Table 5).

**TABLE 5:** Results of the factor analysis for the volition items.

Items		(Lack of) perseverance	Social focus
During practice, I am able to motivate myself even when I’m tired.		**–0.773**	0.092
During practice, I am easily distracted by other things.		**0.676**	0.254
During practice, I tend to put things off a lot.		**0.582**	0.415
When I make a decision during preparation for the contest, I feel confident with it.		**–0.543**	–0.008
Often times during the audition, I think of things completely unrelated to the contest.		**0.410**	0.203
I want to satisfy everyone in the auditions.		0.007	**0.756**
I am reluctant to do hard practice tasks.		0.371	**0.586**
I often fear losing others affection by not participating.		0.050	**0.572**
	Cronbach’s *α*	0.611	0.431
	Variance explained (%)	24.95	19.09

*Extraction method: principal component analysis (PCA). Rotation method: Varimax with Kaiser-Normalization. *KMO*: 0.774, Bartlett: *p* < 0.001. Bold values indicate the highest factor loading for each item.*

Unfortunately, we were unable to test our instrument beforehand due to time constraints.

### Incentives, Flow, and Volition in Correlation With Gender, Amount of Practice, and Contest Category

After having computed the different factors, differences within the samples regarding the participants’ incentives, feelings of flow, and volition were tested and their interdependencies with the participants’ gender, the amount of practice they engaged in, and the category they participated in were explored.

**FIGURE 3:**
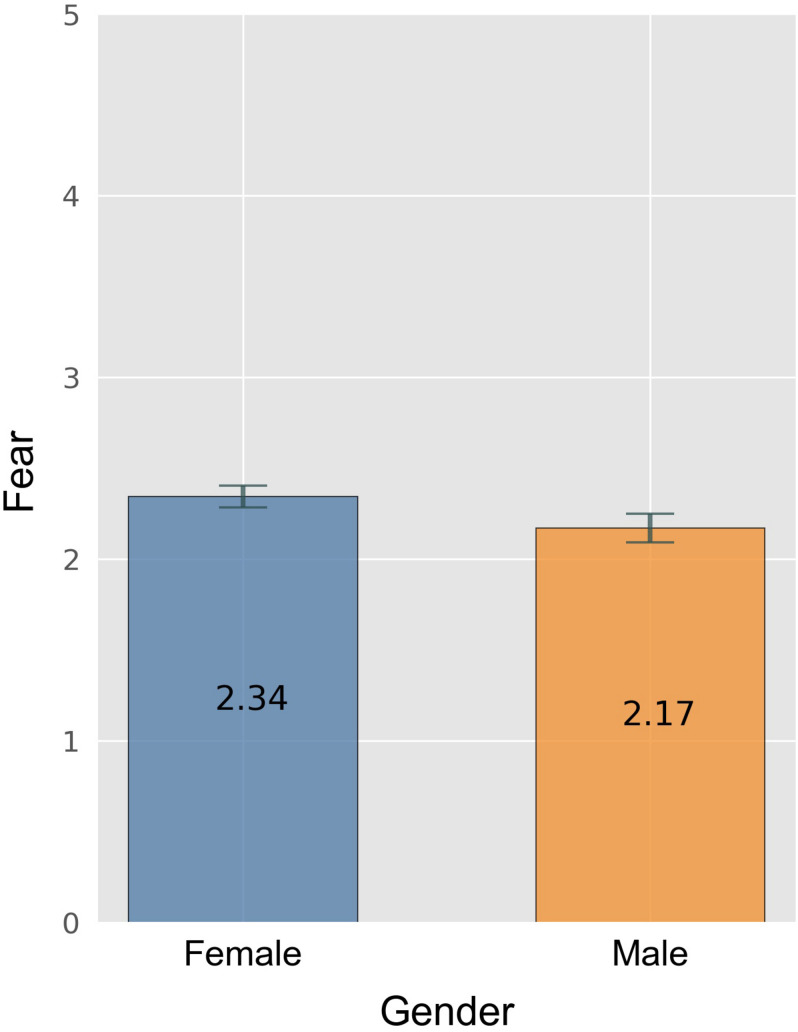
Fear by gender. Error bars indicate 0.95 confidence interval estimations (standard error * 1.96).

Significant gender effects for the motivation scales could only be identified for the scale “fear.” Female participants scored significantly higher on that scale than males (*t*[1114] = 3.43, *p* = 0.001) (cf. Figure 3). All of the following ANOVAS in this chapter were two-factorial ANOVAs with the independent variables “amount of practice” and “contest category”. The respective dependent variable is named in the text.

**FIGURE 4:**
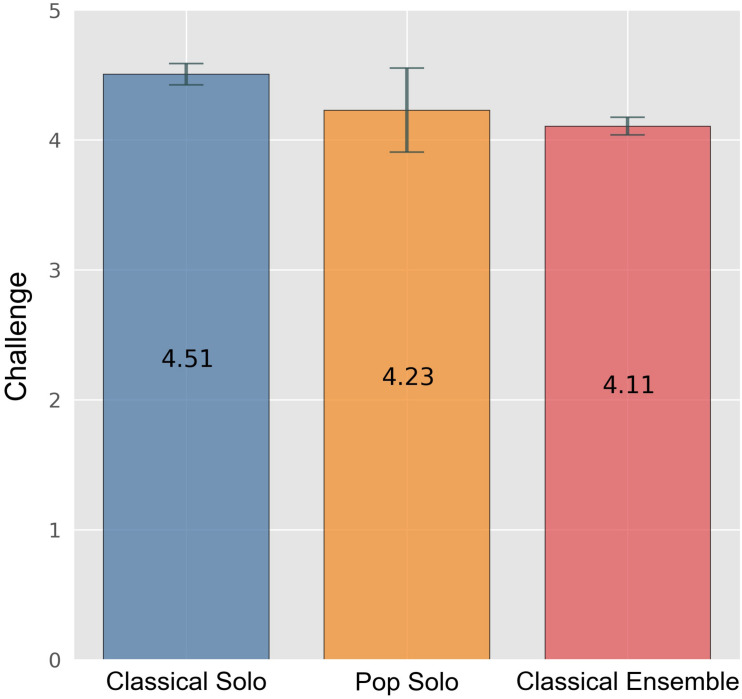
Challenge by contest category. Error bars indicate 0.95 confidence interval estimations (standard error * 1.96).

Nearly the whole sample (86%) agreed or strongly agreed that the incentive “challenge,” one of the single items, was decisive. We could observe that challenge is most important for the participants in the classical solo contest and that they score significantly higher on that item in comparison with participants in the ensemble category (*F*[5,2] = 14.685, *p* < 0.001, $\eta_{\text{p}}^{2}$ = 0.028) (cf. Figure 4). Moreover, participants of the pop solo contest showed significantly more general flow than all other participants (*F*[5,2] = 4.321, *p* = 0.014, $\eta_{\text{p}}^{2}$ = 0.008). Concerning the volition scale “social focus,” only participants in the classical solo contest and participants in the ensemble category differed significantly (*F*[5,2] = 3.803, *p* = 0.023, $\eta_{\text{p}}^{2}$ = 0.007), with ensemble players scoring higher on that scale.

**FIGURE 5:**
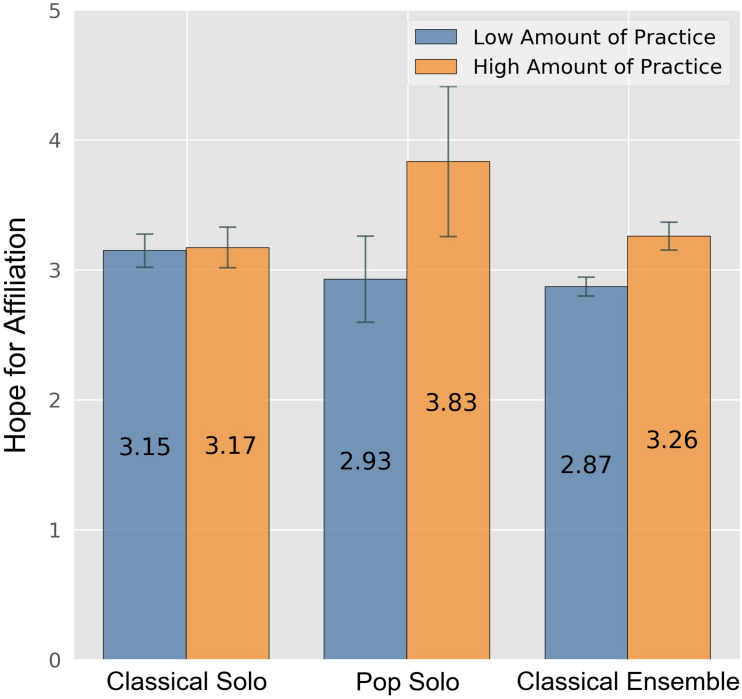
Hope for affiliation by contest category and weekly amount of practice. Error bars indicate 0.95 confidence interval estimations (standard error * 1.96). Participants with more than 8:00 h of practice were assigned a “High Amount of Practice.”

**FIGURE 6:**
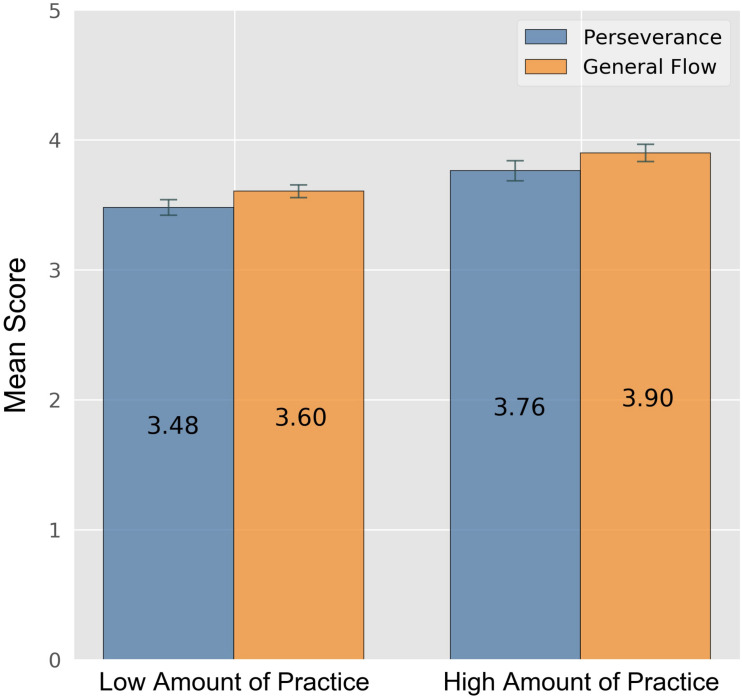
Perseverance and general flow by regular amount of practice. Error bars indicate 0.95 confidence interval estimations (standard error * 1.96). Participants with more than 8:00 h of practice were assigned a “High Amount of Practice.”

There was a significant main effect for the dependent variable “hope for affiliation” for amount of practice (*F*[5,1] = 12.209, *p* < 0.001, $\eta_{\text{p}}^{2}$ = 0.011) and a significant interaction (*F*[5,2] = 5.750, *p* = 0.003, $\eta_{\text{p}}^{2}$ = 0.011): Participants in the pop solo contest were especially driven by hope for affiliation, if they practiced a lot (cf. Figure 5). Although not as pronounced, this was also found for participants in the ensemble contest. For participants in the classical solo contest, no such interaction was identified. Participants who practiced a lot preparing for the contest scored significantly higher on the scale “general flow” than the participants with a smaller amount of practice (*F*[5,1] = 10.393, *p* = 0.001, $\eta_{\text{p}}^{2}$ = 0.010) and also showed significantly more perseverance (*F*[5,1] = 4.807, *p* = 0.029, $\eta_{\text{p}}^{2}$ = 0.005) (cf. Figure 6). One could imagine the flow experiences by adolescents who practice a lot furthered perseverance, but the inverse may also be true. Moreover, participants reporting a low amount of practice scored significantly higher on the scale “social focus” than participants who practiced a lot (*F*[5,1] = 10.07, *p* = 0.002, $\eta_{\text{p}}^{2}$ = 0.009).

**FIGURE 7:**
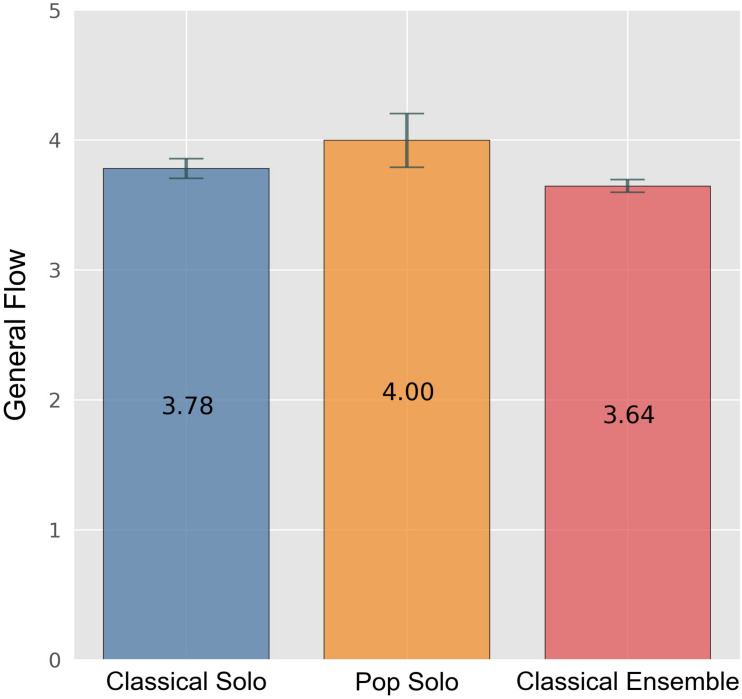
General flow by contest category. Error bars indicate 0.95 confidence interval estimations (standard error * 1.96).

Additionally, in an ANOVA with the dependent variable “general flow” we found main effects for both amount of practice and contest category (*F*[5,1] = 10.393, *p* = 0.001, $\eta_{\text{p}}^{2}$ = 0.010 and *F*[5,2] = 4.321, *p* = 0.014, $\eta_{\text{p}}^{2}$ = 0.008) (cf. Figures 6, 7). Surprisingly, there was no correlation between “amount of practice” and “flow” in individuals participating in the classical solo category.

Furthermore, the participants of the “Jugend musiziert” contest were asked by whom they were motivated to participate in the contest. A descriptive analysis showed that 75 percent were motivated by their instrumental teachers. Still, more than half of the participants (54%) said that they were motivated by themselves to participate. Only 27 percent stated that they were motivated by their parents and 17 percent were motivated by other people like peers, fellow ensemble players, or siblings. There were several differences between contest categories, as displayed in Table 1. It shows that classical solo players were motivated by their parents to the largest degree. Differences in instrumental teachers as a source of motivation were minor, but classical ensemble and pop solo players agreed the most and the least, respectively. Classical solo players regarded themselves as self-motivated much more than participants from the other two contest categories. Twenty percent of classical ensemble players stated another motivational source, which is more than double than the share of the other participants.

### Coherence Between the Scales and Differences Between Participants in the Three Contest Categories

To test the newly built scales for coherence, correlations between the different variables were computed. A significant positive correlation could be found between the scale “general flow” and “perseverance” (*r* = 0.558). In addition, the motivational factor “fear” showed a significant positive correlation with the sub-scale “fear of evaluation” of the MPAI-A-D (*r* = 0.533). Furthermore, significant correlations between “neuroticism” as a personality trait and both “physical anxiety symptoms” (*r* = 0.521) and “fear of evaluation” (*r* = 0.500), as sub-scales of performance anxiety, were found (see Figures 3–5 and Tables 1–3 with descriptive statistics in the Supplementary Material). In terms of content, these correlations were not very astonishing; rather, they confirmed the validity of the newly built scales. For example, adolescents with a fear incentive also presented with a fear of evaluation and emotional instability, which leads to physical anxiety symptoms during their performance.

In order to further investigate the differences between participants in the three contest categories, a linear discriminant analysis (LDA) was conducted. Variables were included in the LDA only if they showed a significant effect on contest category in a preceding Kruskal–Wallis test. This non-parametric test was chosen because meeting the assumptions for ANOVA was questionable due to unequal group sizes and non-normality. In case of doubt, it is safe to use the Kruskal–Wallis test (Kraska-Miller, 2013). The 17 variables in the Kruskal–Wallis test were preselected on the grounds of theory-based assumptions.

**FIGURE 8:**
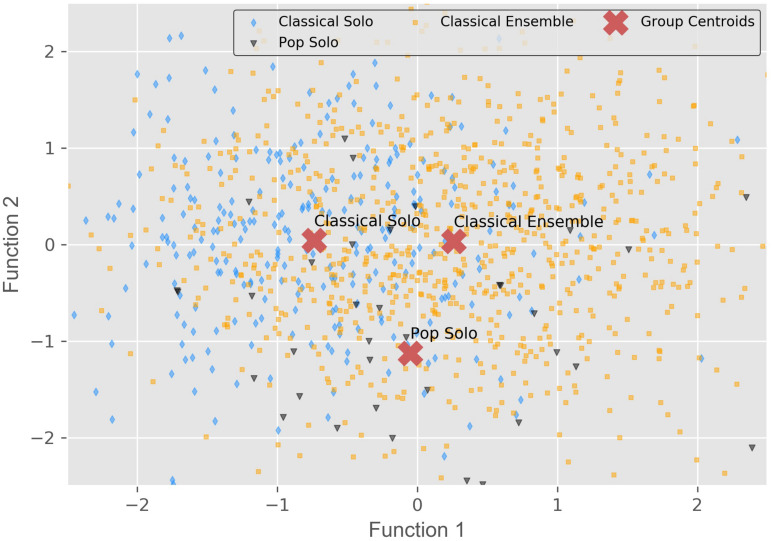
Group centroids for the contest categories located on the two canonical discriminant functions. Method: Linear Discriminant Analysis.

Significant effects (*α* = 0.05) were found for 11 of the 17 tested variables, implying complex differences between categories (cf. Table 6). All significant variables were included in the LDA. The two resulting canonical discriminant functions F1 and F2 show moderate and low discriminant power, respectively (Eigenvalue λ_F1_ = 0.187, λ_F2_ = 0.038, Canonical Correlation Coefficient *r*_F1_ = 0.397, *r*_F2_ = 0.191, Wilks-Lambda Λ_*F1*_ = 0.812, Λ_F2_ = 0.964). However, a Chi-Squared test was significant (*p* < 0.001) for both functions, implying that they can, to some extent, be used to differentiate between the groups. Table 7 shows the standardized canonical discriminant function coefficients for all variables. The overall results are visualized in Figure 8. Function 1 distinguishes between all three categories, however, only to a small degree. It is characterized by high “fear of humiliation” as well as low “challenge” and “concern.” Function 2 separates the pop solo category from both classical categories. Its highest coefficients are “conscientiousness” (–), “general flow” and “neuroticism” (–). While function 1 can be seen as separating participants by group-size (ensemble vs. solo), function 2 predicts genre-affiliation (pop vs. classical).

**TABLE 6:** Results of the Kruskal–Wallis *H* test.

Scales and variables	Kruskal–Wallis *H*	*p*
General flow	13.40	0.001*
Concern	6.37	0.041*
Social focus	13.71	0.001*
Perseverance	12.74	0.002*
Physical anxiety symptoms	5.34	0.069
Fear of evaluation	3.05	0.218
Fear of humiliation	145.64	< 0.001*
Hope for affiliation	7.89	0.019*
Fear incentive	4.44	0.108
Hope for admiration	5.41	0.067
Challenge	39.15	< 0.001*
Extraversion	0.62	0.734
Neuroticism	10.58	0.005*
Openness	10.44	0.005*
Conscientiousness	21.12	< 0.001*
Agreeableness	0.29	0.866
Musical ambition	36.55	< 0.001*

*Significant at α = 0.05.

**TABLE 7:** Standardized canonical discriminant function coefficients for all tested constructs.

	Standardized canonical discriminant function coefficient	
	Function 1	Function 2
Hope for affiliation	−0.029	0.216
Challenge	−0.280	0.098
General flow	0.063	−0.499
Concern	−0.226	−0.167
Fear of humiliation	0.886	−0.085
Perseverance	0.110	−0.136
Social focus	0.133	−0.078
Neuroticism	−0.166	0.312
Openness	−0.037	−0.109
Conscientiousness	0.002	0.752
Musical ambition	−0.094	−0.270

## Discussion

Music contests are an important means of discovering talents and promoting musical abilities. The present paper aims at updating the findings from previous research concerning the music contest “Jugend musiziert,” especially with regard to the amount of practice, instrument-, genre-, age-, and gender-specific practice time and interdependencies between incentives, flow, volition and practice time, which were analyzed in more detail in this article than in earlier studies on “Jugend musiziert.”

Analyzing the data, different incentives, volitional, and flow factors seem to exist in the participants at the highest level of the contest “Jugend musiziert.” Concerning the incentivizing aspects, four different factors were identified that might be important for young contestants facing a challenge like a music contest for highly gifted young musicians. Apart from “fear” as a negatively connoted factor, the positive incentives “hope for affiliation,” “challenge,” and “hope for admiration” were identified. In view of the fact that we only employed a short selection of items concerning the incentives for participation in the contest used in Bullerjahn et al. (2017), the comparability of the results is quite striking: The only negatively connoted incentive in this former study was “fear of rejection” and the positive ones were “hope for affiliation,” “achievement” (to which the item “challenge” belongs) and “power” (with “hope for admiration” as one of the items). All hope components of the three basic psychological needs in MDT-terminology (McClelland, 1985) are represented, but the fear components of all psychological needs together form their own factor. Possibly, this is due to the special situation of the “Jugend musiziert” music competition at the national level, where much is at stake: Winning a first prize could be the pathway to a professional music career, just as a poor performance could end such dreams.

Regarding the volitional aspects that become important during practice as well as during the audition the two factors “perseverance” and “social focus” were identified. While the latter of these two factors may be important especially when playing in an ensemble, the former may apply to situations of ensemble as well as solitary practice. Particularly perseverance might be crucial for the contestants to be able to maintain practicing even if they do not actually want to practice. This could facilitate a transition from a volitional into a motivational mindset during practice, thereby possibly reaching a state of flow (Roth and Sokolowski, 2011).

Studying flow as an incentive while practicing, the data suggest a two-factor solution containing a general flow factor and a second factor expressing the adolescents’ concern when preparing for “Jugend musiziert.” This result is consistent with the findings of Rheinberg et al. (2003), who, when developing the Flow Short Scale, suggested a general flow factor as one possible interpretation of the factorial structure and found an additional factor called “concern.”

The data indicate differences in the amount of practice between the three contest categories. Classical solo players were shown to practice the most, followed by participants of the classical ensemble and pop solo categories. These differences could be explained by the different affordances and settings of practice in the respective categories. While practicing alone or with a maximum of one accompanying player for the classical solo category may be more flexible and individual, practice for participants in the classical ensemble category could possibly be complicated by making appointments for joint practice, resulting in less overall practice time. Instrumental practice of contestants in the pop solo category may not only occur during explicit preparation for the contest, but also in more informal settings, such as jam sessions or band practice. The result that contestants of this category reported less practice time compared to the other two categories could be explained by the participants of the pop solo category not including these informal practice sessions into their answer.

Mean weekly practice time was found to be 9:25 h for participants in the classical solo category. This is substantially lower than results from previous studies concerned with participants at the national level. In those studies, the mean was shown to be about 24 h (Bastian, 1991: national and federal state level of “Jugend musiziert”) and about 11 h of weekly practice (Linzenkirchner and Eger-Harsch, 1995: regional level), respectively, but only about 5 h of weekly practice in Bullerjahn et al. (2017: regional level, solo, and ensemble).^5^ In a dissertation by Kaczmarek (2012), the weekly practice time of highly gifted adolescents at pre-college music programs amounted to almost 20 h, compared with average students at VdM music schools amounting to slightly more than 6 h. Differences could be explained in multiple ways: Perhaps, increased educational demands, resulting from the shortening of pre-tertiary education from 13 to 12 years, might have had an impact. However, it is also possible that instrumental teachers pay more attention to the ways their students practice. It is conceivable that practice strategies have improved, leading to a lower amount of practice time overall. Investigating a possible change in instrumental teaching might be the focus for future research projects.

Regarding motivation, the overall most prominent aspect, namely the need for challenge, was most pronounced for classical solo players. Participants of the solo categories showed higher flow than those in the classical ensemble category, with pop solo players scoring the highest. Ensemble players, however, reported a higher social focus than solo participants. Among pop solo players, the factor hope for affiliation was found to be higher for those who practiced much when compared to those who practiced less.

Aggregating these and other differences between categories, an LDA suggests that it is possible to establish a typology comparing the pop solo, the classical solo, and the classical ensemble category. In addition, it reveals the attributes which are typically exhibited by the participants of the respective categories. Classical solo participants turned out to be low in fear of humiliation, and high in challenge, as well as concern. The opposite applies to the classical ensemble category, although much less distinctively. The difference in fear of humiliation can be explained by the social versus individual context of an ensemble and solo audition, respectively. However, standing in the spotlight alone seems to raise concern, possibly because negative criticism and shame cannot be dampened through ‘shared pain.’ The prototypical participant of the pop solo category is low in conscientiousness, high in general flow, and low in neuroticism. The latter was also highly correlated with performance anxiety and fear incentive. In summary, most of the differences between participants could be found between the three contest categories while there were only minor differences regarding other factors such as gender and age, even though the effect sizes were very small throughout.

Parents and family as “persons in the shadow” provide a rich music-oriented sociotope, which values and intensively supports music activities, so that children are enabled to develop their musical giftedness to excellence (cf. Gembris and Bullerjahn, 2018; see also Lehmann and Kristensen, 2014; Ziegler and Stöger, 2016). Overall, parental support decreased with participants’ age. However, while parents attended fewer concerts, their willingness to spend time and/or money stayed relatively stable. Of course, other aspects, such as general emotional support, may play a role in participants’ perception of their parental support. Thus, it is not entirely permissible to exclude the possibility that it is merely the form of parental support that changed with contestants’ age. Nonetheless, it is interesting to note that parents were viewed as less central to motivation for participation than either instrumental teachers or self-encouragement. This finding lends further support to the conclusion that parent involvement may not be as high as one might presume for some of the contestants. It seems sensible to assume that participants’ independence from their parents and their support and involvement increases with age. Consequently, it is likely that parental support does indeed decrease the older the contestants are. However, they practice more than younger participants, which shows that they are able to activate a great deal of volition when needed. These results are in line with the findings by Davidson et al. (1996). A further examination investigating the way students were motivated by their social environment, including teachers, parents, peers, and others, might be worthwhile. For example, it might be interesting to distinguish between gentle encouragements or more pronounced pressure.

Our findings confirm earlier results of the *Investigating Musical Performance (IMP)* research project with 244 participants (70% of them undergraduates; 55% male; 48% classical, 27% popular, 18% jazz, and 7% Scottish traditional musicians), which suggests that popular, jazz, and folk musicians experience more pleasure in musical activities than their classical counterparts (de Bézenac and Swindells, 2009) and that classical musicians are more prone to performance anxiety (Papageorgi et al., 2011). Furthermore, the latter tended to have begun engaging with music at an earlier age (Creech et al., 2008), were shown to be more influenced by parents (de Bézenac and Swindells, 2009), and demonstrated higher performance skills and quality (Papageorgi et al., 2009). The motivation of non-classical musicians was shown to stem primarily from intrinsic factors (de Bézenac and Swindells, 2009). Additionally, female musicians tended to be less confident and more at risk of having negative performance experiences and suffering from performance anxiety (Papageorgi et al., 2011). Surprisingly enough, there was no statistical evidence of any major interaction between gender and musical genre (Welch et al., 2008).

Naturally, this study is not without its limitations. Firstly, psychometric instruments are often too long and space-consuming to be suitable for questionnaire studies. Thus, shortened and changed versions of standardized instruments were mainly used. For some of these shortened versions, there has been a trade-off between their measurement precision and ease of use. Furthermore, we were unable to pretest the questionnaire beforehand. Therefore, many reliabilities turned out weak or mediocre. Nevertheless, since the study was conducted during the contest itself with the contest being an important aspect in the life of a young musician, it may be more ecologically valid than laboratory studies. Moreover, after the survey we conducted factorial analyses to assure the construct validity of our scales and we calculated correlations with the personality scales of the BFI-10 that confirmed the criterion validity of our instrument.

The different contest categories that were compared in this paper are very broad categories that contain very different instruments, e.g., voice and piano in the classical solo category. This fact as well as the possible age differences in the respective categories may complicate a realistic comparison of practice time. Therefore, in future research, further comparisons between contestants with different instruments would be helpful to achieve a more detailed understanding of differences in practice times. In this paper, only information about practice time as the quantitative part of practice behavior was collected. Although this information holds implications about one’s dedication toward musical practice, qualitative aspects of practice behavior like deliberate practice strategies need to be studied further.

Unfortunately, some of the variables we collected did not show a normal distribution, possibly due to the special sample of highly gifted young musicians. Additionally, it is plausible that many participants had close relatives who also participated in the contest. Therefore, the sample might not be representative for other musical contests or highly gifted musicians in general. Because of this unusual sample, the results of this study cannot easily be transferred to other samples. Testing our results for a less special sample could include further studies on participants of the regional and federal state level of “Jugend musiziert.” Furthermore, by computing multiple ANOVAs, the results could be influenced by higher alpha errors. The questionnaire was very long (17 pages); perhaps a shorter one would have resulted in more, and more attentive, participants. Furthermore, there are large differences in sample sizes between the contest categories. Lastly, participants were restricted to certain instruments according to the call for contest.

## Conclusion

In conclusion, this study points to a number of implications that need to be reconsidered carefully. Our findings have highlighted the fact that there are major differences between participants of different contest categories. This is of special interest in the face of former fierce arguments concerning the opening of “Jugend musiziert” to popular music participants (Grunenberg, 2003; Braun, 2007). Our findings also confirm that motivation is critical for sustained practice, and therefore for taking on the challenges which go hand in hand with the preparation for a music contest, especially one like “Jugend musiziert,” in which participants need to compete and succeed at three different levels. It is, however, beyond the scope of this study to evaluate if these results are only valid for German participants of a music youth contest or for music contests in general. Further research could try to integrate the observation of rehearsal strategies used by the contestants to broaden the insight into the practice behavior of highly gifted young musicians.

Additional results of this particular large-scale study will be published in Gembris and Bullerjahn (in preparation). Further research on the “Jugend musiziert” contest could focus on possible psychosocial risk factors for the young participants, such as problematic sibling constellations, overeager parents, and burnout as a result of personal perfectionism, as well as on their musical preferences and their use of media. Role models and career aspirations could be other possible topics for further research.

## Data Availability Statement

The datasets presented in this article are not readily available because the data are property of Paderborn University. Requests to access the datasets should be directed to heiner.gembris@uni-paderborn.de.

## Ethics Statement

The study was conducted in full accordance with the *Ethical Guidelines* of the German Association of Psychologists (DGPs) and the German Association of Psychologists (BDP) as well as the *Ethical Principles of Psychologists and Code of Conduct* of the American Psychological Association (APA). These guidelines suggest that for the type of research reported here, a formal ethics approval is not necessary. This is due to the fact that the study only made use of completely anonymous questionnaires and thus, no identifying information was obtained from the participants. Moreover, participants and their parents were informed about the aim of the questionnaire, the anonymity of the data, and that participation was voluntary. In accordance with the ethical principles mentioned above, it was not required to obtain written informed consent.

## Author Contributions

HG, CB, JD, and JM conceived and designed the questionnaire and performed the survey. CB, JD, and MH analyzed the data. CB, JD, MH, and CK wrote the manuscript. JM contributed to the calculations and passages from a presentation script. All authors contributed to the article and approved the submitted version.

## Conflict of Interest

The authors declare that the research was conducted in the absence of any commercial or financial relationships that could be construed as a potential conflict of interest.

## Footnotes

1 All ensembles of the “Jugend musiziert” contest consist of 2–5 players and are not conducted. The category “Neue Musik” (Contemporary Music) represents an exception. In this category, one ensemble can consist of up to 13 young musicians and the use of additional tape recordings is allowed. These ensembles can also be conducted, but the conductor needs to be an adolescent as well.

2 Technically, the competition is for years 12 and older. However, younger students are allowed to compete as part of an ensemble. Hence, a small number of participants (*n* = 25) are younger than twelve (see Supplementary Figure 1).

3 Excluding participants who might have been too young to handle the questionnaire properly (<12 years) did not change the results noticeably or in a meaningful way.

4 Practice time includes both solitary practice and practice with accompanists or ensembles.

5 It is rather difficult to compare the studies on “Jugend musiziert” because they all use different methodologies (e.g., retrospective surveys, interviews etc.) and samples (e.g., different levels, categories etc.). However, because of the limited number of studies available, it was decided to include them nonetheless.
