# Supplementary material for: Why Adolescents Participate in a Music Contest and Why They Practice – The Influence of Incentives, Flow, and Volition on Practice Time
Source: Front Psychol. 2020 Oct 27;11:561814. doi: 10.3389/fpsyg.2020.561814 (PMC7652895; doi:10.3389/fpsyg.2020.561814)
Supplement: Supplementary file 1 [file Data_Sheet_1.pdf]

Supplementary Material

1 Supplementary Figures

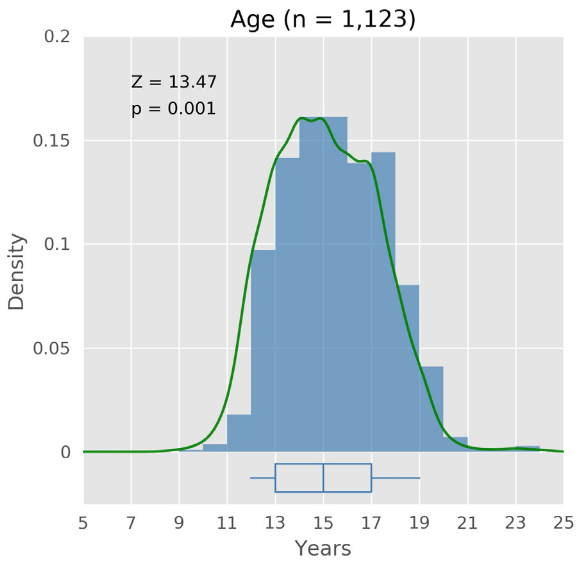

Supplementary Figure 1. Age Distribution. Histogram, Kernel Density Estimation (KDE), Boxplot (Whiskers indicate the 5th and 95th percentiles), and D'Agostino & Pearsons test of normality (D'Agostino, R., and Pearson, E. S. (1973). Tests for departure from normality. *Biometrika*, 60, 613-622).

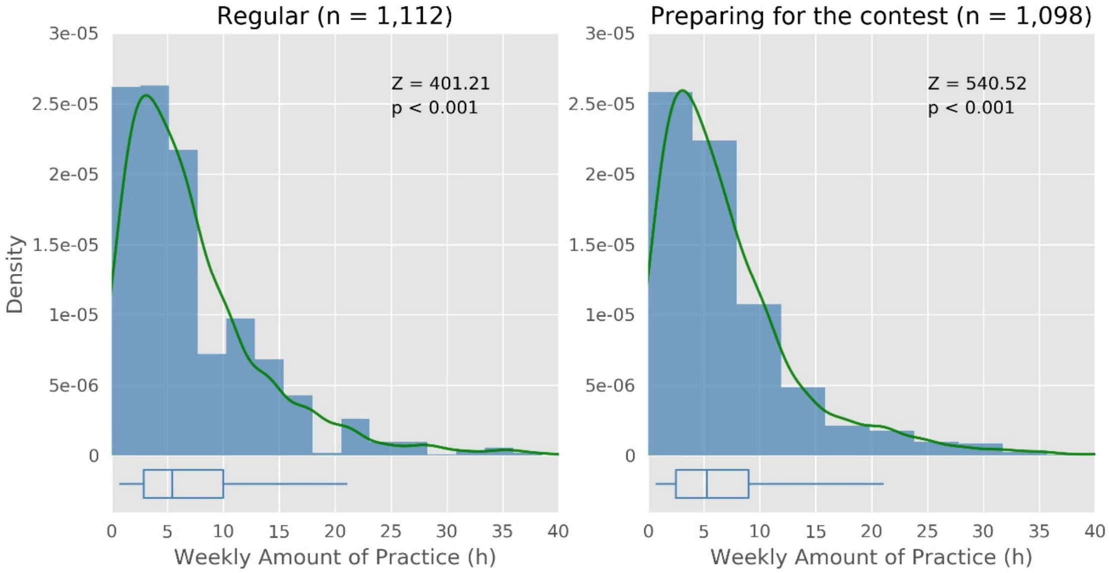

Supplementary Figure 2. Distribution of the Amount of Practice. Histogram, Kernel Density Estimation (KDE), Boxplot (Whiskers indicate the 5th and 95th percentiles), and D'Agostino & Pearsons test of normality (D'Agostino, R., and Pearson, E. S. (1973). Tests for departure from normality. *Biometrika*, 60, 613-622).

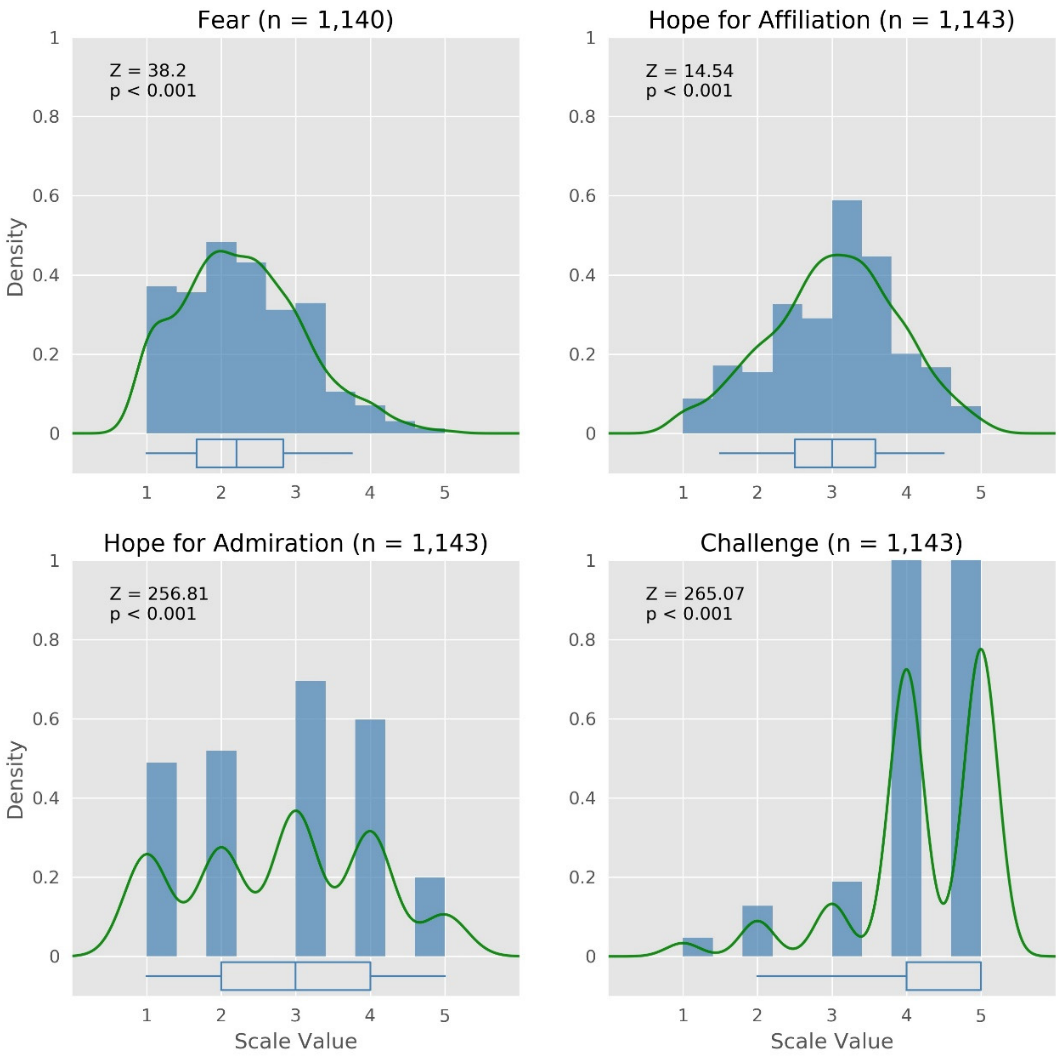

Supplementary Figure 3. Distribution of the Motivation Scales. Histogram, Kernel Density Estimation (KDE), Boxplot (Whiskers indicate the 5th and 95th percentiles), and D'Agostino & Pearsons test of normality (D'Agostino, R., and Pearson, E. S. (1973). Tests for departure from normality. *Biometrika*, 60, 613–622).

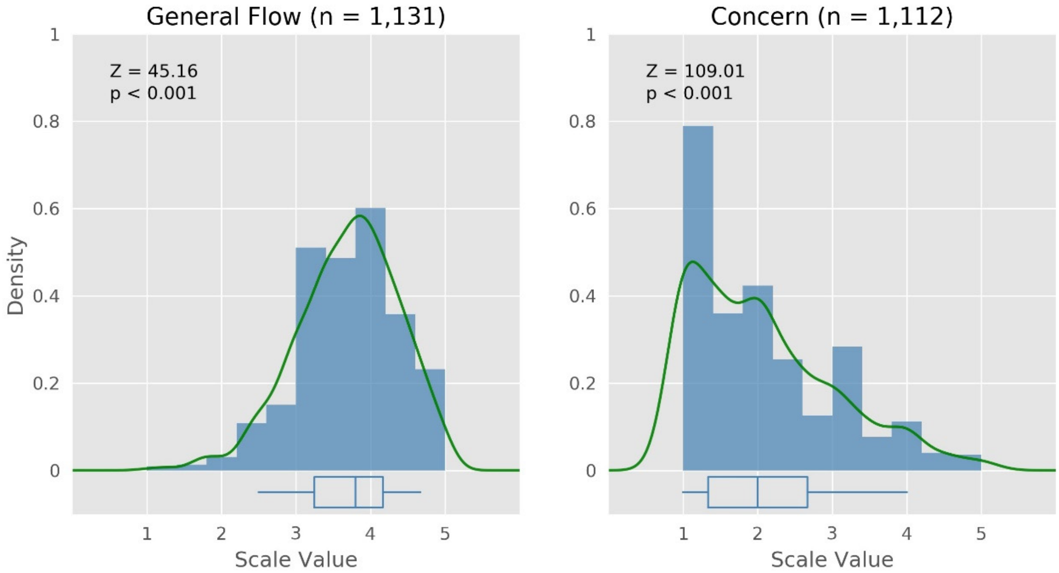

Supplementary Figure 4. Distribution of the Flow Scales. Histogram, Kernel Density Estimation (KDE), Boxplot (Whiskers indicate the 5th and 95th percentiles), and D'Agostino & Pearsons test of normality (D'Agostino, R., and Pearson, E. S. (1973). Tests for departure from normality. *Biometrika*, 60, 613–622).

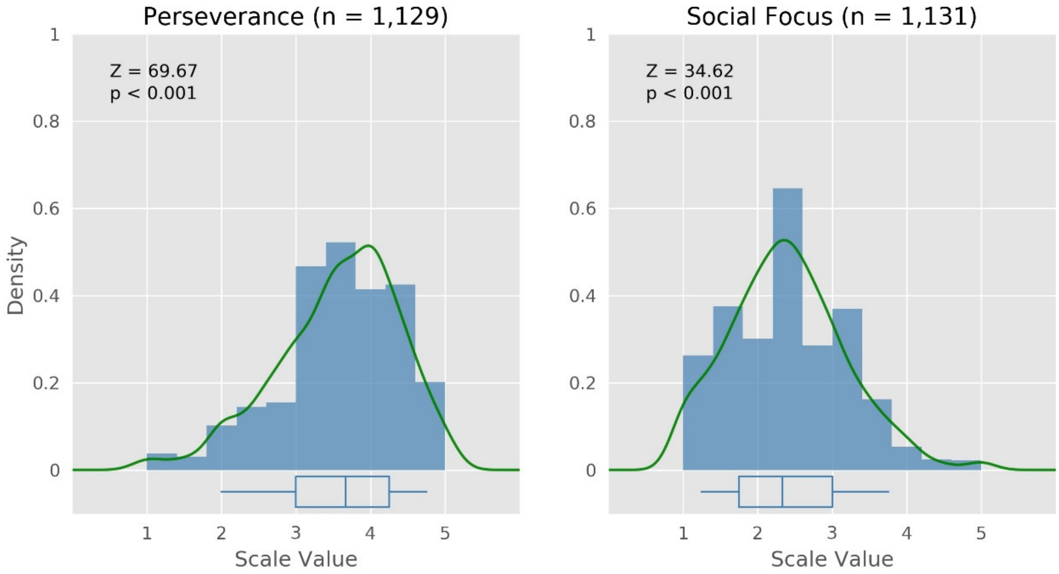

Supplementary Figure 5. Distribution of the Volition Scales. Histogram, Kernel Density Estimation (KDE), Boxplot (Whiskers indicate the 5th and 95th percentiles), and D'Agostino & Pearsons test of normality (D'Agostino, R., and Pearson, E. S. (1973). Tests for departure from normality. *Biometrika*, 60, 613–622).

2 Supplementary Tables

Supplementary Table 1. Personality trait scores by contest category and gender. Items scores range from 1 to 5.

Trait		Contest Category			Gender	
		**Classical Solo**	**Pop Solo**	**Classical Ensemble**	**Female**	**Male**
Openness	Mean	4.15	4.12	3.92	4.01	3.93
	SD	0.84	0.83	0.97	0.96	0.90
Conscientiousness	Mean	3.37	2.55	3.16	3.33	3.00
	SD	1.06	0.94	1.05	1.03	1.05
Extraversion	Mean	3.32	3.46	3.37	3.39	3.32
	SD	1.11	1.05	1.09	1.12	1.04
Agreeableness	Mean	3.17	3.26	3.13	3.20	3.08
	SD	0.92	0.94	0.91	0.91	0.91
Neuroticism	Mean	2.72	2.26	2.74	2.87	2.46
	SD	1.02	1.05	1.01	1.03	0.93

Supplementary Table 2. Performance anxiety scores by category and gender. Items scores range from 1 to 5.

Factor		Contest Category			Gender	
		**Classical Solo**	**Pop Solo**	**Classical Ensemble**	**Female**	**Male**
Physical Anxiety Symptoms	Mean	3.27	2.86	3.20	3.33	3.02
	SD	0.82	0.90	0.91	0.88	0.86
Fear of Evaluation	Mean	2.32	2.15	2.34	2.41	2.18
	SD	0.81	0.85	0.81	0.82	0.76
Fear of Humiliation	Mean	2.03	2.69	3.05	2.83	2.72
	SD	0.98	1.11	1.18	1.24	1.15

Supplementary Table 3. Results of the factor analysis for the German version of the Music Performance Anxiety Inventory for Adolescents (MPAI-A-D).

Item	Physical Anxiety Symptoms	Fear of Evaluation	Fear of Humiliation
I get nervous right before my performance.	**0.807**	0.078	0.004
When I play/sing in front of an audience, my heart starts to beat fast.	**0.759**	0.197	0.138
Before my performance, I get a strange feeling in my belly.	**0.739**	0.271	–0.049
Before my performance, I start to shake.	**0.700**	0.314	0.118
When I play/sing in front of an audience, I am scared to make mistakes.	**0.550**	0.530	0.223
When I play/sing in front of an audience, my hands get sweaty.	**0.505**	–0.016	0.397
I fear that my parents or my teacher will dislike my performance.	0.142	**0.672**	0.090
I often doubt whether I am good enough to perform.	0.425	**0.649**	0.183
After my performance, I am usually satisfied with it.	–0.051	**0.630**	–0.048
When I make a mistake during my performance, I easily start to panic.	0.286	**0.615**	0.267
I prefer to play alone rather than in front of other people.	0.255	**0.517**	0.384
When I play/sing in front of an audience, it's hard for me to focus on the music.	0.311	**0.511**	0.261
I would prefer to play in a group rather than alone.	–0.004	0.094	**0.842**
I try to avoid playing solo at a concert.	0.048	0.264	**0.789**
My muscles are tense when I perform.	0.406	0.111	**0.421**
Cronbach's α	0.813	0.754	0.735
Variance explained (%)	22.91	18.43	13.89

Note: Extraction method: Principal Component Analysis (PCA). Rotation method: Varimax with Kaiser-Normalization.KMO: 0.904, Bartlett: $p < 0.001$.
